# Correlation Between Sarcopenia and Oral Health in Patients on Chronic Hemodialysis

**DOI:** 10.3390/life15050823

**Published:** 2025-05-21

**Authors:** Petra Kovačević Totić, Iva Klarić Puđa, Karla Kovačević Čorak, Velimir Altabas, Milan Milošević, Selma Cvijetić Avdagić, Karmela Altabas

**Affiliations:** 1 University Hospital Centre Sisters of Charity, 10 000 Zagreb, Croatia; 2 Health Center, Zagreb Center, 10 000 Zagreb, Croatia; 3 Croatian Institute of Public Health, 10 000 Zagreb, Croatia; 4 University Hospital Centre Sisters of Charity, School of Medicine, University of Zagreb, 10 000 Zagreb, Croatia; 5 Andrija Stampar School of Public Health, School of Medicine, University of Zagreb, 10 000 Zagreb, Croatia; 6 Institute for Medical Research and Occupational Health, 10 000 Zagreb, Croatia; 7 University Hospital Centre Sisters of Charity, School of Dental Medicine, University of Zagreb, 10 000 Zagreb, Croatia

**Keywords:** sarcopenia, hemodialysis, oral health, SarQoL, OHIP-14

## Abstract

Patients on hemodialysis have signs of chronic systemic inflammation and a higher incidence of sarcopenia. Poor oral health can also trigger systemic inflammation and thus affect sarcopenia. The study included 100 patients on chronic hemodialysis who underwent oral status, routine laboratory measurements, bioimpedance analysis, hand grip strength measurement, and two questionnaires regarding oral health and sarcopenia. Data were analyzed using the Fisher exact test and the Mann–Whitney U test. In total, 28.0% of the participants had sarcopenia. The median total number of erupted teeth in all patients was 12.0 (7.0–23.0). A positive correlation of the total number of erupted teeth and erupted premolars with sarcopenia was confirmed (*p* = 0.035). CRP was significantly elevated in patients with sarcopenia (*p* = 0.035). Laboratory parameters showed that the blood albumin level was reduced in all patients (*p* = 0.002). The median overall score of the SarQoL questionnaire for all participants was 60.37 (43.87–70.61), which indicates that patients on hemodialysis are aware of their limitations caused by sarcopenia. Moreover, SarQoL was significantly negatively correlated with sarcopenia. This study confirmed poorer oral health in hemodialysis patients who had sarcopenia. Therefore, intervention studies are needed to improve the oral health of patients on HD, which could possibly influence the incidence of sarcopenia.

## 1. Introduction

Sarcopenia is an age-related disease defined by a combination of low muscle mass, low muscle strength, and physical performance, which are used to determine the severity of sarcopenia [1,2]. Public health awareness of sarcopenia is increasing, given that it has unfavorable outcomes such as disability, low quality of life, and increased risk of death [3,4,5]. On the other hand, the prevalence of sarcopenia in hospitalized elderly patients is as high as 76% [6]. In hemodialysis patients, there is more intense muscle deterioration compared to patients in the 4th stage of chronic kidney disease [7]. The prevalence of sarcopenia is also significantly higher in hemodialysis patients, so it varies from 9.5 to 37.1% [8,9,10,11]. Reduced muscle mass, whether measured by serum creatinine or body composition analysis, was independently associated with lower survival rates in hemodialysis patients [8,12]. Further research has shown that muscle mass in dialysis patients is inversely correlated with IL-6 and CRP blood levels [13,14]. Chronic inflammation could therefore underlie the hypercatabolic state in dialysis patients.

In addition to chronic inflammation, numerous metabolic disorders that occur in CKD lead to increased protein catabolism, which ultimately results in reduced muscle mass and function, regardless of age [15]. Multifactorial causes that contribute to the development of sarcopenia in patients with CKD include immunological and hormonal changes, changes at the muscle cell level, metabolic acidosis, reduced protein intake, and reduced physical activity [15]. CKD is a catabolic state that leads to the activation of the renin–angiotensin system, which leads to reduced regulation of phospho-Akt and activation of caspase-3 in skeletal muscles. As a result, actin splitting and increased myocyte apoptosis occur [16].

Observational studies have confirmed frequent oral problems in hemodialysis patients, especially periodontal disease, an inflammatory disease that affects the supporting tissues of the teeth [17,18,19]. The most common are plaque-induced gingivitis and periodontitis [20], which ultimately lead to tooth loss.

Other conditions in HD patients that cause poor oral health include uremic stomatitis, gingival bleeding due to platelet dysfunction or anticoagulant therapy, enamel hypoplasia, and tooth surface erosion due to gastric acid regurgitation and vomiting caused by uremia and an increased risk of bone fractures [21]. Dialysis patients often have dry mouth due to water restriction and/or hypofunction of the salivary glands, which is a common side effect of certain medications. Since saliva washes the oral cavity, hyposalivation leads to the accumulation of food debris, which can be a breeding ground for infections [22]. Ruospo et al. noted that hemodialysis patients had a higher incidence of tooth loss and poorer oral hygiene, resulting in more periodontitis compared to CKD patients not on dialysis [23].

Hatta et al. published a review in 2021 regarding the impact of oral health on sarcopenia. It was concluded that there are indications of a connection between the aforementioned conditions, but additional longitudinal studies with precisely defined sarcopenia and oral status are needed [24].

The primary objective of the study was to examine oral health and the presence of sarcopenia in patients on chronic hemodialysis, while the secondary objective was to investigate whether there is a significant correlation between poor oral health and sarcopenia in patients on chronic hemodialysis.

## 2. Materials and Methods

### 2.1. Participants

All patients of both genders who met the inclusion criteria and did not have exclusion criteria (*n* = 100) and who were on a chronic hemodialysis program at the Department of Nephrology and Dialysis of the Clinic for Internal Diseases of the Clinical Hospital Center of the Sisters of Charity and at the dialysis center of the Avitum Polyclinic participated in this research.

Exclusion criteria: age < 18 years, amputated limb, implanted pacemaker or subdiaphragmatic electrical device, prescribed immunosuppressive therapy, and acute illness in the last month.

Inclusion criteria: age > 18 years, time spent on hemodialysis longer than 6 months.

The sample size was calculated using power analysis with 80% power and a significance level of 5%.

The research was conducted after the approval of the Ethics Committee of the School of Dental Medicine, University of Zagreb, and the Clinical Hospital Centre Sisters of Charity, in accordance with the Declaration of Helsinki. All participants signed an informed consent form before the start of the study.

Based on the inclusion and exclusion criteria, the selected participants underwent an oral status assessment, body composition analysis with a bioimpedance device, hand grip strength measurement with a dynamometer, and monitoring of routine laboratory findings that were done every two months during hemodialysis (CRP, albumins).

In addition, we monitored the participants’ demographic indicators (age, gender), body mass, height, body mass index (BMI), duration of dialysis in months, and comorbidities (Charlson index *). Finally, the subjects completed two questionnaires: one related to oral health (OHIP-14) and the other related to sarcopenia (SarQoL).

* The Charlson comorbidity index [25] is a method of categorizing patient comorbidities based on diagnostic codes of the International Classification of Diseases (ICD).

### 2.2. Oral Status

Oral status was determined by a dentist during the hemodialysis procedure. Oral status was assessed using a dental mirror, probe, and light, which included determining the number and position of remaining teeth, the presence of prosthetic replacements, and an assessment of oral hygiene [26], which refers to a set of practices and habits aimed at maintaining the cleanliness of the oral cavity in order to prevent the accumulation of dental plaque, the development of caries, and periodontal diseases. It includes regular tooth brushing, interdental cleaning, and routine dental check-ups.

The oral status included questions about oral symptoms (ammonia breath, dry mouth (subjective), decreased salivation (objective), burning sensation of the oral mucosa, painful sensitivity of the mucosa, altered taste (metallic taste), and the last visit to the dentist. You can find the example of a dentist check-up list in Supplement S1.

The decayed, missing, and filled teeth (DMFT) index was used to assess the prevalence and severity of dental caries in permanent teeth. It is calculated by summing the number of decayed, missing (due to caries), and filled teeth and then dividing by the number of individuals examined [27]. The examination follows standardized WHO methods using a dental mirror and probe.

The oral mucosa was also examined, and the current location of mucosal changes was recorded according to the modified WHO oral mucosal topographic map [28] (Supplement S2).

Changes in the oral mucosa that were included in the examination are the following: pale mucosa, mucosal hematomas, petechiae and ecchymoses, gingival bleeding, generalized erythema (stomatitis), partial erythema (e.g., palatitis prosthetica), mucosal erosions, ulcerations, pseudomembranes, gingivitis, periodontitis, glossitis (exfoliative), candidiasis (white sloughing deposits), hyperkeratosis, lichenoid changes, impaired wound healing, and prolonged bleeding. The presence of enlarged salivary glands, increased plaque formation, caries [27], enamel hypoplasia, and enamel erosion were also examined.

### 2.3. Body Composition Analysis

Body composition analysis was performed using the BIA-ACC^®^ (Biotekna. Venice, Italy) device, which uses multi-frequency currents, including high-frequency analysis up to 1000 kHz (1 MHz), enabling more accurate and comprehensive body composition assessments compared to standard BIA devices [29]. The analysis was performed in the supine position, with arms and legs extended. Two electrodes were applied to the dorsum of the right hand and two to the dorsum of the right foot.

The following parameters were measured: appendicular lean soft tissue (ALST), fat-free mass (FFM), fat mass (FM), intramuscular adipose tissue (IMAT), skeletal muscle mass expressed as % of total body weight (wSMI), and hSMI expressed as skeletal muscle mass (kg) divided by total body height (m^2^).

### 2.4. Grip Strength

An assessment of hand grip strength was performed using a hydraulic dynamometer (Hydraulic Hand Dynamometer SAEHAN SH5001; Rehaforum Medical, Changwon-si, Republic of Korea) prior to hemodialysis in a sitting position on a chair, during which the upper leg and lower leg formed a right angle. The upper arm and forearm of the examined hand were also at the right angle. At the examiner’s signal, the participant had to squeeze the dynamometer as hard as he could for a few seconds, and then rest for 30 s. The dynamometer measurement was repeated three times on each hand.

Based on the results of hand grip strength and body composition analysis, it was concluded which patients had sarcopenia, using the criteria of the European Working Group on Sarcopenia in Older People (EWGSOP2) [1].

### 2.5. Questionnaires

The participants completed the Oral Health Impact Profile OHIP-14 [30,31] and Sarcopenia Quality of Life (SarQoL) [32,33,34] questionnaires, which provided their subjective perceptions of oral health and quality of life regarding sarcopenia.

### 2.6. Data Analysis

Data are presented in tables and graphics. The data were prepared using a Microsoft Office Excel version 2016 MSO (16.0.4266.1001) spreadsheet calculator. The distribution of continuous numerical values was analyzed using the Kolmogorov–Smirnov test, and corresponding non-parametric tests were applied to the obtained data. Categorical and nominal values are presented through corresponding frequencies and proportions, and differences are analyzed using Fisher’s exact test. Continuous values are presented through medians and interquartile ranges, and differences between independent groups are analyzed using the Mann-Whitney U test and are shown by a Box and Whisker plot, in which median values, interquartile ranges, minimum and maximum values, and extreme values that differ from the median by more than 1.5 interquartile ranges are shown. *p* Values less than 0.05 are considered significant. Licensed program support IBM SPSS Statistics, version 25.0 (https://www.ibm.com/analytics/spss-statistics-software- accessed on 27 March 2024), was used for the analysis.

## 3. Results

Descriptive statistics of the examined clinical and sociodemographic categorical variables for all included subjects are shown in Table 1. Of the total number of subjects (*N* = 100), 28 (28.0%) had sarcopenia. The majority of the participants were male (61.0%). Almost 50% of the participants, 46 of them (46.0%), last visited a dentist several years ago. In total, 39 (39.0%) participants had poor oral hygiene, and 52 (52.0%) had satisfactory oral hygiene. There were 37 (37.0%) participants without dental prosthesis.

Descriptive statistics of the sociodemographic and anthropometric variables observed as continuous values in all subjects are shown in Table 2. The median age was 68.5 (60.0–78.0) years, and the body mass index was 26.12 (23.06–30.89) kg/m^2^.

Table 3 shows descriptive statistics of laboratory and clinical variables related to oral health that were observed as continuous values in all subjects. The OHIP-14 questionnaire scores were 12.0 (4.0–17.75), while the total SarQoL score was 60.37 (43.87–70.61).

**Figure 1:**
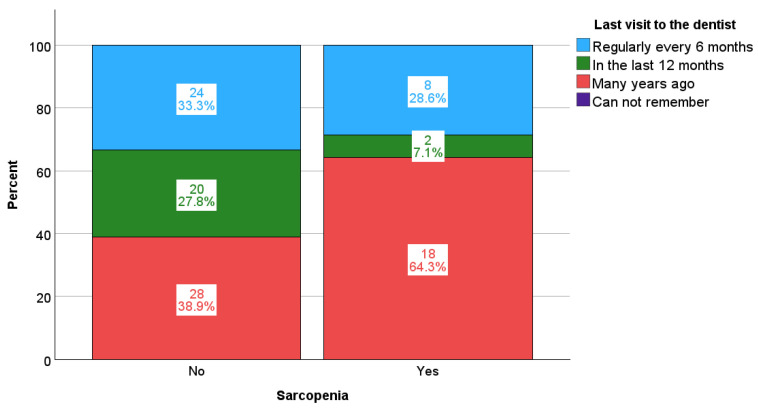
Differences in the frequency of dental visits according to the presence of sarcopenia: *p* = 0.030.

Differences in the frequency of the examined clinical and sociodemographic categorical variables with respect to the presence of sarcopenia are shown in Table 4. The only significant differences related to the time of the last visit to the dentist, with participants who had sarcopenia visiting the dentist significantly less frequently; i.e., 18 of them (64.3%) stated that they had last visited the dentist several years ago, while respondents who did not have sarcopenia confirmed this in 38.9% of cases; *p* = 0.030 (Figure 1).

Differences in anthropometric parameters according to the presence of sarcopenia are shown in Table 5. As expected, the group with sarcopenia was significantly older (*p* < 0.001) and had significantly higher values of total body fat (*p* = 0.047), higher IMAT expressed as a percentage (*p* < 0.001), lower wSMI (*p* = 0.001), lower hSMI (*p* = 0.008), lower proportion of striated muscle (*p* = 0.003), as well as weaker grip of the dominant and non-dominant hand (*p* < 0.001).

Differences in laboratory and clinical variables related to oral health according to the presence of sarcopenia are shown in Table 6. Albumin concentration was significantly lower in subjects with sarcopenia (*p* = 0.002), while CRP levels were significantly higher (*p* = 0.035), but still within the clinical reference ranges that do not indicate an acute inflammatory process (>5 mmol/L). ALST was significantly more negative in the sarcopenia group (*p* = 0.003). There were no significant differences in the OHIP-14 values, nor in the Charlson index, while in SarQoL the differences were significant in domains D1 (*p* = 0.003), D2 (*p* = 0.002), D3 (*p* = 0.042), D4 (*p* < 0.001), D5 (*p* < 0.001) and D7 (*p* < 0.001), in which, as a rule, lower values were in the group with sarcopenia. The total SarQoL score was significantly lower in the group with sarcopenia: 43.63 (30.10–60.56) versus 62.78 (49.83–74.24); *p* < 0.001.

**Figure 2:**
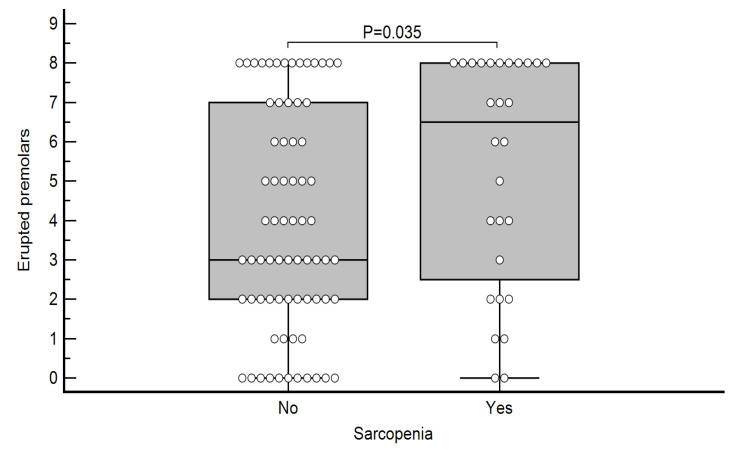
Differences in the number of erupted premolars according to the presence of sarcopenia.

**Figure 3:**
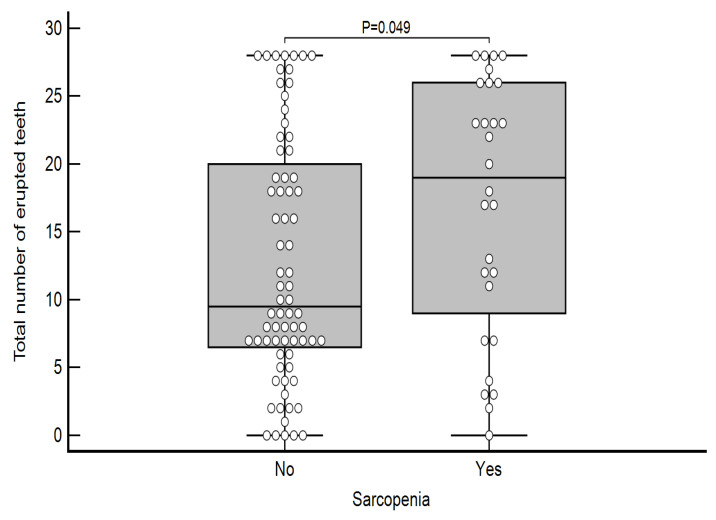
Differences in the total number of erupted teeth according to the presence of sarcopenia.

Differences in the number of erupted teeth regarding the presence of sarcopenia are shown in Table 7. Subjects with sarcopenia had a significantly higher number of lost premolars (*p* = 0.035; Figure 2), as well as a total number of lost teeth (*p* = 0.049; Figure 3).

## 4. Discussion

In this study, we confirmed that 28.0% of the total number of subjects (N = 100) had sarcopenia (Table 1), while in other studies, it was shown that the proportion of patients with end-stage CKD and sarcopenia was around 37% in men and 29% in women [9]. A meta-analysis of the world prevalence of sarcopenia conducted in 2017 pointed out that the prevalence of sarcopenia in healthy older people was about 10% in men and about 10% in women [35]. The majority of the subjects in our study were male (61.0%) (Table 1).

Poor oral hygiene was recorded in as many as 39.0% of the subjects, and 46.0% of all subjects last visited a dentist several years ago (Table 1). It is interesting to point out that 64.3% of the patients with confirmed sarcopenia last visited a dentist several years ago, while in those without sarcopenia, the share was 38.9% (Table 4, Figure 1). The reason for the infrequent visits to the dentist in patients with sarcopenia could be poor mobility and physical functional capacity, along with the frequent occurrence of depression in patients on HD [36,37], which may be why they often give up going to the dentist.

A positive correlation between the total number of lost teeth and lost premolars with sarcopenia was confirmed (Table 7, Figure 2 and Figure 3). This can be explained in two ways. One is a poorer ability to chew, form a bolus, and swallow [38], especially meat, due to partial or total toothlessness, which ultimately results in a reduced protein intake that favors the development of sarcopenia [39]. However, it is also important to note that in most cases, the premolars and molars, which have the main function in chewing solid substances, had fallen out. Another explanation for sarcopenia and missing teeth is the fact that due to poor oral hygiene and uremic conditions, chronic inflammation occurs in the periodontium, which favors the development of periodontitis [40], which later results in tooth loss, and chronic inflammation from the oral cavity spreads systemically, which could contribute to the worsening of sarcopenia. Patients with chronic kidney disease have a high prevalence of periodontal disease, including gingivitis and periodontitis, which are associated with the progression of CKD through chronic inflammation [20,41]. It is important to emphasize that this chronic inflammation state in periodontitis has significant implication on cardiovascular diseases [42,43], as well, causing possible worsening and higher mortality [44,45,46].

Kaizu et al. confirmed that in patients on HD, there is a negative correlation between elevated inflammatory parameters (IL-6, CRP) and muscle mass, which was measured by cross-section of the thigh on CT [12]. In our study, CRP was significantly elevated in patients with sarcopenia (*p* = 0.035) (Table 6), in contrast to those without sarcopenia (3.45 vs. 2.70), and the stated elevated value of CRP could indicate the presence of chronic inflammation.

Among the laboratory parameters, the albumin blood level was analyzed, which had decreased in all subjects (*p* = 0.002). However, in patients with sarcopenia, the values were lower than in patients without sarcopenia (37.50 vs. 40.15 g/L) (Table 7). Thus, the aforementioned patients with sarcopenia had, in addition to hypoalbuminemia, poorer oral health with a greater number of lost teeth, resulting in the potential inability to get enough protein through food, which results in hypoalbuminemia and, in the long term, sarcopenia. Chen et al. thus confirmed the correlation between severe periodontitis and hypoalbuminemia in 253 patients on hemodialysis [16]. It is known that the level of albumin decreases in inflammatory diseases due to the blocking of pro-inflammatory cytokines in the production of hepatic albumin [47]. On the other hand, Cholewa et al. found no difference in albumin levels between dentate and edentulous patients on HD or patients with healthy periodontium or gingivitis and those with periodontitis [13].

**Figure 4:**
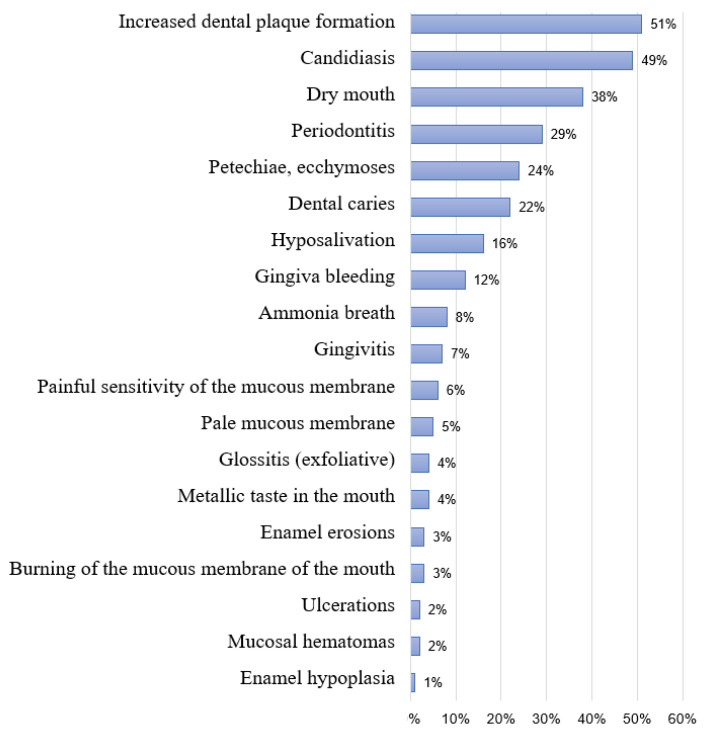
Symptoms and signs related to oral health in all participants (*N* = 100).

It has been observed that patients with CKD often experience various oral signs and symptoms, such as xerostomia, ammonia-like breath, metallic taste in the mouth, mucosal damage, oral mucosal malignancies, and oral infections such as candidiasis [48]. In our study, 51% of the subjects had increased calculus formation, 49% had oral candidiasis, 38% had periodontitis, as many as 24% had petechiae on the mucosa, and 22% had dental caries (Figure 4). In total, 8% of the subjects had ammonia-like breath, and 4% had a metallic taste in the mouth Increased calculus formation in 51% of the subjects is consistent with previous studies, most likely due to changes in salivary composition due to impaired calcium and phosphorus metabolism and increased urea levels in saliva, which increase salivary pH, thereby increasing the risk of calculus development [49]. Ammonia breath is caused by poor oral hygiene, increased urea concentrations in saliva, and its conversion to ammonia [50], while increased phosphate levels and changes in salivary pH result in a metallic taste in the patient’s mouth [51].

Dry mouth was experienced by 38% of the subjects, and possible causes include fluid restriction in patients with CKD, hyposalivation, mouth breathing, and the use of certain antihypertensive medications. Xerostomia is therefore a predisposing factor for the development of candidiasis, bacterial infections, caries, and periodontal disease due to the loss of the protective effect of saliva [51,52]. In addition to xerostomia, risk factors for the development of candidiasis include poor oral hygiene, diabetes, age, total dentures, and hyposalivation [53].

As many as 49% of our respondents had candidiasis, which is a very high percentage, and coincides with the research conducted by Yeter in 2019 [54]. In that study, candidiasis was confirmed in 53% of the patients on hemodialysis, in addition to which hsCRP and IL-6 were elevated, while HDL was significantly lowered.

This points to the important role of oral candidiasis in chronic inflammation and the development of atherosclerotic disease of blood vessels [54]. Considering that candidiasis also promotes systemic inflammation and that chronic inflammation is the basis of sarcopenia, candidiasis could also be a potential factor for the worsening of sarcopenia in HD patients.

**Figure 5:**
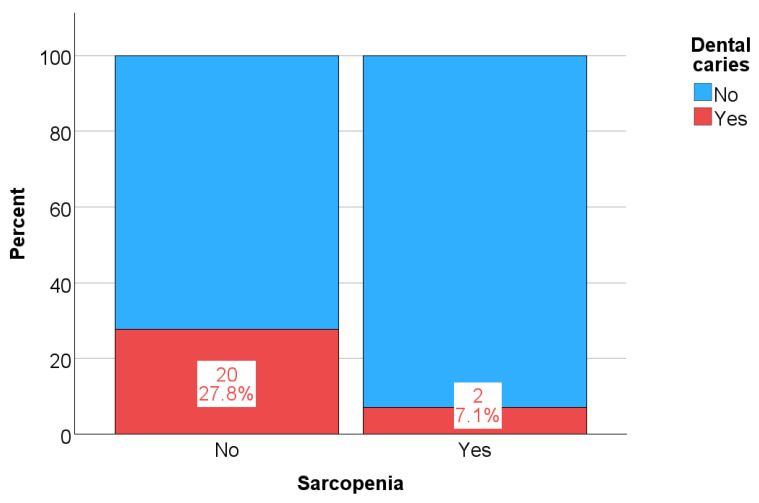
Differences in caries incidence according to the presence of sarcopenia: *p* = 0.031.

Of all the examined signs and symptoms of oral health, significant differences were recorded only in the frequency of dental caries, which was significantly more frequent in the group that did not have sarcopenia (*p* = 0.031, Figure 5). Thus, only 7% of the patients with sarcopenia had confirmed caries, possibly due to the smaller total number of teeth in patients with sarcopenia. Another reason for the lower incidence of caries in patients on HD who have sarcopenia could be the longer dialysis vintage in patients with sarcopenia—61 vs. 39 months (Table 6)—which is why these patients are exposed to the state of uremia for a longer time, and thus the urea in saliva achieves a stronger antimicrobial effect, which is why the mentioned patients have less caries.

In this study, in patients with sarcopenia, there was no positive correlation with dentition and the presence of dental restorations, possibly due to the need for a larger number of participants. However, it should be noted that in patients with sarcopenia, total edentulousness was present in 17.9% of the patients, in contrast to 9.7% in patients who did not have sarcopenia (Table 4), i.e., of the total number of subjects, 12% had total edentulousness (Table 1).

In a systematic review that included 11,340 subjects in the last stage of CKD, Ruospo et al. presented results in which 20% of the subjects on dialysis had total edentulousness [23], while in our case, it was 12%. Ruospo emphasizes the danger of a combination of oral diseases, chronic inflammation, and consequent malnutrition, which leads to a potential risk for the development of cardiovascular complications and a higher mortality rate in patients with end-stage CKD [23].

The comorbidities of all patients were approximately the same, which is confirmed by the Charlson median comorbidity index in patients with sarcopenia, as well as in those without sarcopenia, which amounted to 6 points.

In our study, body composition was analyzed using a dual-frequency bioimpedance device that provides some of the parameters for confirming the diagnosis of sarcopenia (ALST or hSMI). However, the device also measures other body composition parameters that may be indirectly related to sarcopenia, such as intramuscular fat tissue (IMAT), fat tissue mass (FM), fat-free body mass (FFM), skeletal muscle expressed as a % of total body mass (wSMI), and skeletal muscle expressed as a % of total body height (hSMI). Almost all of the above parameters in this study correlated with sarcopenia (Table 5), which also further confirms the validity of the diagnostic parameters for diagnosing sarcopenia according to EWGSOP2 [1].

An important predictor of muscle atrophy and the subsequent development of sarcopenia is IMAT, which is defined as the accumulation of ectopic adipose tissue between muscle groups under the muscle fascia and intramuscular adipose tissue, which is visible on magnetic resonance (MR) images [55]. The BIA-ACC device, which we used to measure bioimpedance, had the option of measuring IMAT, which is much more practical and economical than using MR.

In our participants, we confirmed a significant correlation between IMAT and sarcopenia (Table 5), but it is necessary to emphasize the importance of IMAT as a possible predictor of sarcopenia, considering that sarcopenia is preceded by muscle infiltration with fatty tissue. Therefore, future research should investigate its role in this sense, with the aim of preventing sarcopenia.

Given that the grip strength of the dominant hand is one of the diagnostic criteria for establishing a diagnosis of sarcopenia (men < 27 kg, women < 16 kg) [28], in our research we confirmed a significant correlation between the grip strength of the dominant hand and sarcopenia, and the grip strength of the non-dominant hand was significantly reduced (Table 5). Heimbürger et al., in their research related to the determination of markers for patient nutrition, confirmed that hand grip strength could be a reliable and economical way to determine and monitor the nutritional status of patients with CKD, given that it significantly negatively correlates with patients who have malnutrition and lean body mass [56]. Moreover, handgrip strength is known to be a strong predictor of all-cause mortality risk in dialysis patients [57].

An important aspect of this study is the subjective perception of hemodialysis patients regarding their oral health and sarcopenia. The oral health questionnaire is the OHIP-14, which covers 7 different domains of daily life with its 14 questions [30,58].

The median total score for all subjects was 12 (4–17.75) (Table 3), while the median score for patients with sarcopenia was 13.50, in contrast to patients without sarcopenia, where it was 12.0 (Table 6).

The total score of the OHIP-14 questionnaire did not achieve a significant correlation with sarcopenia. Such results indicate that hemodialysis patients consider their oral health satisfactory, given that the maximum score is 56 points, and a higher score indicates poorer oral health.

The above subjective opinion of the participants about their own oral health did not coincide with the clinical findings, which confirmed that poor oral hygiene is present in as many as 39% of the subjects, with partial edentulism in 80% of the cases (Table 1). In 2016, Schmalz et al. also confirmed that the clinical status of the teeth does not correlate with the subjective opinion about oral health in patients on hemodialysis or after kidney transplantation [59]. The reason for this is difficult to determine, but it could be assumed that the patients became accustomed to poor oral health over time, given that such a condition progresses slowly, and adjusted their diet accordingly. On the other hand, since these are patients with severe and chronic diagnoses, it is possible that they do not burden themselves with oral health, i.e., do not consider it to be so threatening to their general health.

In contrast to the unrealistically good opinion about their own oral health, the hemodialysis patients in our study had a more realistic opinion about their quality of life according to sarcopenia. The median total score of the SarQoL questionnaire for all subjects was 60.37 (43.87–70.61) (Table 3), and given that out of a maximum of 100 points, a lower score indicates a worse quality of life, it can be concluded that hemodialysis patients are aware of their problems and limitations caused by sarcopenia. Moreover, SarQoL was significantly negatively correlated with sarcopenia in our patients, which confirms the precision of the questionnaire used. Thus, the median total score in patients with sarcopenia was significantly lower and was 43.63 (30.10–60.56) compared to 62.78 (49.83–74.24) in patients who did not have sarcopenia (Table 6). All domains of the SarQoL questionnaire (physical and mental health—D1, movement—D2, body composition—D3, functionality—D4, activities of daily living—D5, fears—D7), except domain D6 (leisure activities), reached a significant negative association with sarcopenia (Table 6).

As in our study, Beaudart et al., in a study of 296 subjects, confirmed a significant association of the SarQoL questionnaire with patients with sarcopenia, which further demonstrates that the questionnaire is valid and reliable and can therefore be recommended for clinical and research purposes. However, its sensitivity to changes needs to be assessed in future longitudinal studies [32].

This research has certain limitations. First, there was a relatively small number of participants, which was limited by the inclusion and exclusion criteria and the patient’s willingness to participate in the research. Another limitation is the lack of a control group.

In contrast, the positive side of this research is its originality in establishing a new connection between oral status and sarcopenia in the population of hemodialysis patients, who are a very specific and sensitive group of patients. It is important to emphasize that this research used a multi-frequency bioelectrical impedance device that measures body composition in detail, including intramuscular adipose tissue (IMAT), which until now has been most often assessed via CT or MR. Thus, the method of measuring body composition in this research is more ethically acceptable and economical. It is a great advantage that two dialysis centers were included, but future research should include more centers. Moreover, all the dialysis centers of the city of Zagreb could contribute to more precise research with a larger number of participants.

### Future Directions

This study is one of the few that has confirmed the correlation of a correctly diagnosed sarcopenia (EWGSOP 2 criteria) with poor oral health, given that the underlying cause of both conditions is chronic inflammation, which opens up numerous questions for new research. Given that this was a cross-sectional study on a relatively small number of subjects, further longitudinal follow-up randomized studies with a larger number of participants are needed. Our study confirmed that patients rarely go for dental check-ups and do not practice adequate oral hygiene; therefore, it is necessary to further investigate whether the oral health of patients on HD differs in other parts of Croatia, as well as in other countries, which may depend on the habits and culture of individual nations, and accordingly decide how to change patients’ habits with the aim of improving oral hygiene. Likewise, intervention studies are needed to monitor the effect of improving oral status and sarcopenia on cardiovascular mortality, which is significantly increased in patients on HD. The best way to improve sarcopenia is to perform therapeutic resistance exercises during the HD process with the introduction of high-protein enteral supplements, which, in combination with dental intervention, should yield promising results (better nutritional status of the patient, greater functional capacity, lower risk of falls, and reduced cardiovascular mortality).

## 5. Conclusions

This study confirmed that hemodialysis patients who suffer from sarcopenia also have poorer oral health, specifically the greater total number of erupted teeth and the total number of erupted premolars, in comparison to patients without sarcopenia. However, it is important to say that HD patients were quite satisfied with their oral health, which was in opposition to their clinical status. On the contrary, their subjective opinion about quality of life regarding sarcopenia showed that those who had sarcopenia were severely limited in most domains of the SarQol questionnaire. Therefore, intervention studies are needed to improve the oral health of hemodialysis patients, which could potentially affect the lower incidence and severity of sarcopenia.

## Figures and Tables

**Table 1:** Descriptive statistics of the examined clinical and sociodemographic categorical variables in all included subjects (*N* = 100).

		*N*	%
Sarcopenia	No	72	72.0%
	Yes	28	28.0%
Gender	Male	61	61.0%
	Female	39	39.0%
Last visit to the dentist	Regularly every 6 months	32	32.0%
	During last 12 months	22	22.0%
	Several years ago	46	46.0%
	Can’t remember	0	0.0%
Oral hygiene	Poor	39	39.0%
	Satisfactory	52	52.0%
	Good	8	8.0%
	Excellent	1	1.0%
Dentition	All teeth present	8	8.0%
	Partial dentition	80	80.0%
	Total edentulousness	12	12.0%
Dental restorations	No dental restorations	37	37.0%
	Total prosthesis	17	17.0%
	Partial prosthesis	7	7.0%
	Dental bridge on one jaw	17	17.0%
	Dental bridge on both jaws	5	5.0%
	Combination	4	4.0%
	Total + partial prosthesis	13	13.0%

**Table 2:** Descriptive statistics of sociodemographic and anthropometric variables and body composition observed as continuous values in all participants (*N* = 100).

	Min	Max	Centile		
			25th	Median	75th
Age (years)	21.00	93.00	60.00	68.50	78.00
Weigh (kg)	43.50	122.00	65.00	75.75	89.00
Height (cm)	146.00	198.00	162.00	169.00	176.00
BMI (kg/m^2^)	16.80	72.00	23.06	26.12	30.89
FFM %	45.00	81.00	60.00	66.00	71.00
FFM kg	28.00	77.60	44.00	49.55	55.53
FM %	19.00	55.00	29.00	34.00	40.00
FM kg	8.70	64.90	18.95	25.05	35.38
IMAT %	1.20	3.50	2.13	2.50	2.80
IMAT kg	0.60	4.10	1.50	1.90	2.30
wSMI %	11.00	31.00	18.00	22.50	26.00
hSMI kg/m^2^	2.50	10.50	4.65	6.10	7.10
Skeletal muscle %	18.20	45.50	29.05	34.70	38.30
Skeletal muscle kg	6.00	35.30	12.90	17.55	20.90
Hand grip of the dominant hand (kg)	6.00	48.00	19.38	25.65	31.53
Hand grip of the non-dominant hand (kg)	0.00	47.60	15.60	20.30	26.60

BMI = body mass index, FFM = fat-free mass, FM = fat mass, IMAT = intramuscular adipose tissue expressed as % of total body weight, wSMI = skeletal muscle mass expressed as % of total body weight (kg), hSMI = skeletal muscle mass expressed as skeletal muscle mass (kg) divided by total body height (m^2^).

**Table 3:** Descriptive statistics of laboratory and clinical variables related to oral health that were observed as continuous values in all subjects (*N* = 100).

	Min	Max	Centile		
			25th	Median	75th
Albumin (g/L)	10.90	46.80	37.18	39.70	42.45
CRP (mg/L)	0.30	86.50	1.38	3.10	9.23
ALST (kg)	5.80	30.60	11.78	15.80	18.70
Dialysis vintage (months)	6.00	460.00	24.00	43.00	84.00
Charlson index	2.00	14.00	5.00	6.00	8.00
OHIP-14	0.00	33.00	4.00	12.00	17.75
SarQoL D1	27.77	99.97	47.77	59.97	73.03
SarQoL D2	13.89	100.00	38.89	55.56	65.98
SarQoLl D3	29.17	100.00	41.67	50.00	66.67
SarQoL D4	13.46	100.00	44.23	65.38	83.62
SarQoL D5	0.00	100.00	30.69	51.67	70.00
SarQoL D6	0.00	83.12	33.25	33.25	33.25
SarQoL D7	62.50	100.00	75.00	87.50	100.00
SarQoL in total	22.65	96.30	43.87	60.37	70.61

CRP = C-reactive protein, ALST = appendicular lean soft tissue, OHIP-14 = Oral Health Impact Profile-14, SarQoL = Sarcopenia Quality of Life.

**Table 4:** Differences in frequencies of examined clinical and sociodemographic categorical variables with regard to the presence of sarcopenia: Fisher’s exact test.

		Sarcopenia				*p*
		Yes *N* = 72		No *N* = 28		
		*N*	%	*N*	%	
Gender	Male	44	61.1%	17	60.7%	0.971
	Female	28	38.9%	11	39.3%	
Last visit to the dentist	Regularly every 6 months	24	33.3%	8	28.6%	0.030
	In the last 12 months	20	27.8%	2	7.1%	
	Many years ago	28	38.9%	18	64.3%	
	Can’t remember	0	0.0%	0	0.0%	
Oral hygiene	Poor	27	37.5%	12	42.9%	0.760
	Satisfactory	39	54.2%	13	46.4%	
	Good	5	6.9%	3	10.7%	
	Excellent	1	1.4%	0	0.0%	
Dentition	All teeth present	6	8.3%	2	7.1%	0.496
	Partial dentition	59	81.9%	21	75.0%	
	Toothlessness	7	9.7%	5	17.9%	
Dental prostheses	Without prostheses	27	37.5%	10	35.7%	0.443
	Total prostheses	12	16.7%	5	17.9%	
	Partial prostheses	5	6.9%	2	7.1%	
	Bridge on one jaw	15	20.8%	2	7.1%	
	Bridge on both jaws	4	5.6%	1	3.6%	
	Combined prostheses	2	2.8%	2	7.1%	
	Total + partial prostheses	7	9.7%	6	21.4%	

**Table 5:** Differences in anthropometric parameters according to the presence of sarcopenia: Mann–Whitney U test.

Sarcopenia		Min	Max	Centile			*p*
				25th	Median	75th	
Age (years)	No	21.00	93.00	52.25	66.00	72.00	<0.001
	Yes	52.00	91.00	69.25	78.50	81.00	
BMI (kg/m^2^)	Ne	17.90	72.00	22.67	25.88	31.21	0.902
	Da	16.80	35.09	23.68	26.43	30.85	
FFM %	No	45.00	81.00	60.00	68.00	71.75	0.069
	Yes	53.00	79.00	59.25	63.00	67.75	
FM %	No	19.00	55.00	28.00	32.00	39.75	0.047
	Yes	21.00	47.00	32.25	37.00	40.75	
IMAT %	No	1.20	3.50	2.10	2.40	2.70	<0.001
	Yes	2.30	3.20	2.50	2.75	2.90	
wSMI %	No	11.00	31.00	19.00	23.50	26.75	0.001
	Yes	13.00	26.00	16.00	20.00	22.75	
hSMI kg/m^2^	No	2.50	10.50	5.10	6.30	7.10	0.008
	Yes	3.00	8.10	4.23	5.30	6.25	
Skeletal muscle %	No	18.20	45.50	30.78	35.90	38.85	0.003
	Yes	19.40	39.30	25.83	30.95	35.08	
Hand grip of dominant hand	No	12.00	48.00	23.00	28.00	34.45	<0.001
	Yes	6.00	26.00	12.38	17.45	21.83	
Hand grip of non-dominant hand	No	0.00	47.60	17.70	24.30	30.00	<0.001
	Yes	4.00	26.00	12.00	15.60	17.60	

BMI = body mass index, FFM = fat-free mass, FM = fat mass, IMAT = intramuscular adipose tissue, wSMI = skeletal muscle mass expressed as % of total body weight (kg), hSMI = skeletal muscle mass expressed as skeletal muscle mass (kg) divided by total body height (m^2^).

**Table 6:** Differences in laboratory and clinical variables with regard to the presence of sarcopenia: Mann–Whitney U test.

Sarcopenia		Min	Max	Centile			*p*
				25th	Median	75th	
Albumin (g/L)	No	10.90	46.80	38.08	40.15	43.10	0.002
	Yes	29.30	43.40	35.10	37.50	40.30	
CRP	No	0.30	86.50	1.18	2.70	7.40	0.035
	Yes	0.60	57.00	1.93	3.45	27.85	
ALST (kg)	No	5.80	30.60	12.75	17.00	19.58	0.003
	Yes	7.00	19.90	10.53	13.30	16.15	
Dialysis vintage (months)	No	6.00	208.00	20.00	39.00	71.00	0.072
	Yes	9.00	460.00	32.00	61.00	94.75	
Charlson index	Ne	2.00	14.00	4.00	6.00	8.00	0.110
	Da	3.00	11.00	6.00	6.00	9.00	
OHIP-14	No	0.00	29.00	3.25	12.00	16.75	0.214
	Yes	2.00	33.00	7.00	13.50	21.00	
SarQol D1	No	27.77	99.97	49.43	64.42	76.63	0.003
	Yes	27.77	85.53	34.43	49.99	61.93	
SarQol D2	No	13.89	100.00	41.67	58.33	69.44	0.002
	Yes	13.89	97.22	25.70	43.06	57.64	
SarQol D3	No	29.17	100.00	41.67	54.17	66.67	0.042
	Yes	29.17	79.17	41.67	50.00	57.29	
SarQol D4	No	32.69	100.00	57.69	73.08	86.78	<0.001
	Yes	13.46	94.64	31.73	44.23	64.08	
SarQol D5	No	11.67	100.00	38.34	58.63	73.33	<0.001
	Yes	0.00	75.00	19.11	41.67	55.42	
SarQol D6	No	0.00	83.12	33.25	33.25	33.25	0.070
	Yes	0.00	66.50	16.62	33.25	33.25	
SarQol D7	No	62.50	100.00	87.50	87.50	100.00	<0.001
	Yes	62.50	100.00	62.50	75.00	87.50	
SarQol in total	No	24.35	96.30	49.83	62.78	74.24	<0.001
	Yes	22.65	83.24	30.10	43.63	60.56	

**Table 7:** Differences in the number of erupted teeth according to the presence of sarcopenia: Mann–Whitney U test.

Sarcopenia		Min	Max	Centile			*p*
				25th	Median	75th	
Erupted incisors	No	0.00	8.00	0.00	1.00	4.75	0.063
	Yes	0.00	8.00	0.25	4.00	8.00	
Erupted canines	No	0.00	4.00	0.00	0.00	2.00	0.117
	Yes	0.00	4.00	0.00	1.50	3.00	
Erupted premolars	No	0.00	8.00	2.00	3.00	7.00	0.035
	Yes	0.00	8.00	2.25	6.50	8.00	
Erupted molars	No	0.00	8.00	4.00	6.00	8.00	0.061
	Yes	0.00	8.00	5.00	7.50	8.00	
Total number of erupted teeth	No	0.00	28.00	6.25	9.50	20.50	0.049
	Yes	0.00	28.00	8.00	19.00	26.00	

## Data Availability

The data that support the findings of this study are available from the corresponding author, [P.K.T.], upon reasonable request.

## Supplementary Materials

The following supporting information can be downloaded at: https://www.mdpi.com/article/10.3390/life15050823/s1, Supplement S1: Example of a dental check-up list; Supplement S2: Modified World Health Organization topographic map of the oral mucosa.
