# Supplementary material for: Correlation Between Sarcopenia and Oral Health in Patients on Chronic Hemodialysis
Source: Life (Basel). 2025 May 21;15(5):823. doi: 10.3390/life15050823 (PMC12113631; doi:10.3390/life15050823)
Supplement: Supplementary file 1 [file life-15-00823-s001.zip › life-3585145-supplementary.pdf]

ORAL STATUS:

PATIENT: ________________

- Availability of dental treatment:

- last visit to the dentist: (regularly every 6 months) (12 months ago) (several years ago)

(does not remember the last time he/she was at the dentist)

Oral hygiene: poor satisfactory good excellent

Dentition: partial dentition total edentulousness

Dental restorations: prothesis total partial

jaw bridges: one jaw both jaws

combined work (mobile and fix))

ORAL SIMPTOMS

Ammonia breath	YES	NO
Dry mouth (subjectively)	YES	NO
Hyposalivation(objektively)	YES	NO
Burning of the mucous membrane of the mouth	YES	NO
Painful mucosal membrane	YES	NO
Metallic taste in the mouth	YES	NO

**ORAL MUCOSA CHANGES**	Yes (+) / No(-)	Localization of lesions according to WHO topography
Pale mucosal membrane		
Mucosal hematoma		
Petechiae, ecchymoses		
Gingiva bleeding		
Generalized erythema (stomatitis)		
Oartial mucosal erythema		
Mucosal erosions		
Ulcerations		
Pseudomembranes		
Gingivitis		
Periodontitis		
Glossitis (exfoliative)		
Candidiasis		
Mucosal hyperkeratosis		
Lichenoid lesions		
Enlargement of the salivary glands		
Increased dental plaque formation		
Dental caries		
Enamel hypoplasia		
Enamel erosions		
Impaired mucosal wound healing		
Prolonged bleeding		

	18	17	16	15	14	13	12	11	21	22	23	24	25	26	27	28
	48	47	46	45	44	43	42	41	31	32	33	34	35	36	37	38

Supplement 1. Example of a dental check-up list.

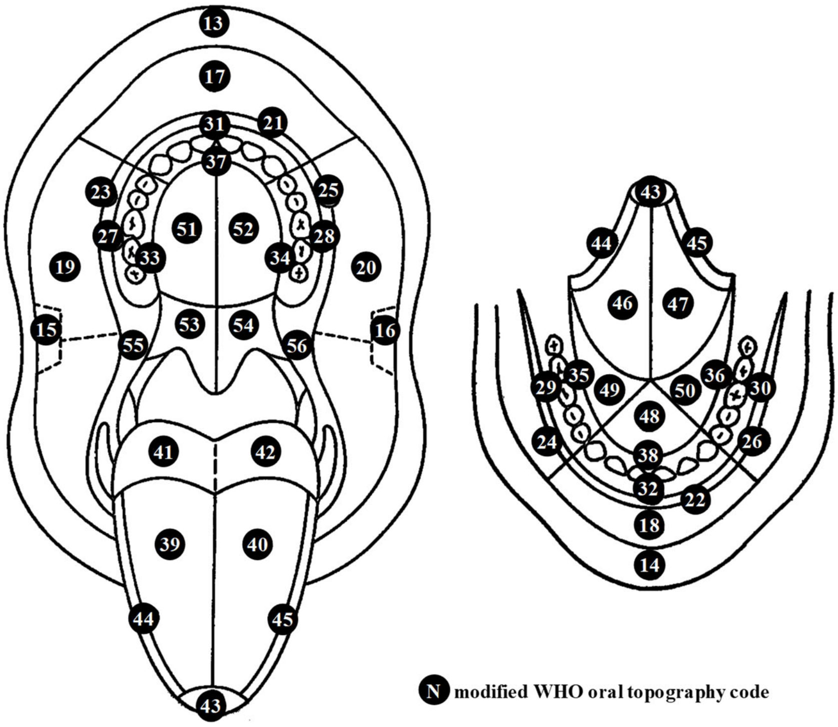

Vermilion border - upper (13), lower (14), labial commissures - right (15), left (16), lip mucosa - upper (17), lower (18), labial sulci - upper (21), lower (22), cheek (buccal mucosa) - right (19), left (20), buccal sulcus - right upper (23) lower (24), buccal sulcus - left upper (25) lower (26), upper gingiva or edentulous alveolar ridge buccally - right (27), left (28), lower gingiva or edentulous alveolar ridge - right (29), left (30), upper anterior gingiva and edentulous ridge labially (31), lower anterior gingiva or edentulous ridge labially (32), upper posterior gingiva or edentulous alveolar ridge palatally - right (33), left (34), lower posterior gingiva or edentulous alveolar ridge lingually - right (35), left (36), anterior gingiva or edentulous ridge palatal (37), and lingual (38), dorsum of tongue - right (39), left (40), base of tongue - right (41), left (42), tip of tongue (43), margin of tongue - right (44), left (45), surface of tongue - right (46), left (47), frontal floor of mouth (48), lateral floor of mouth - right (49), left (50), hard palate - right (51), left (52), soft palate - right (53), left (54), anterior tonsillar column - right (55), left (56).

Supplement 2. Modified World Health Organization topographic map of the oral mucosa.
